# Altered individual behavioral and EEG parameters are related to the EDSS score in relapsing-remitting multiple sclerosis patients

**DOI:** 10.1371/journal.pone.0219594

**Published:** 2019-07-15

**Authors:** Manuel Vázquez-Marrufo, Alejandro Galvao-Carmona, Rocio Caballero-Díaz, Monica Borges, Maria Dolores Paramo, Maria Luisa Benítez-Lugo, Juan Luis Ruiz-Peña, Guillermo Izquierdo

**Affiliations:** 1 Experimental Psychology Department, Faculty of Psychology, University of Seville, Spain; 2 Department of Psychology, Universidad Loyola Andalucía, Seville, Spain; 3 Multiple Sclerosis Unit, Virgen Macarena Hospital, Seville, Spain; 4 Physiotherapy Department, Faculty of Nursing, Physiotherapy and Chiropody, Universidad de Sevilla, Sevilla, Spain; 5 Multiple Sclerosis Unit, Hospital Vithas-NISA, Seville, Spain; Universita degli Studi di Roma La Sapienza, ITALY

## Abstract

Functional neuroanatomy of cognitive impairment in multiple sclerosis is currently still a challenge. During the progression of the disease, several cognitive mechanisms deteriorate thus diminishing the patient’s quality of life. A primary objective in the cognitive assessment of multiple sclerosis (MS) patients is to find reliable measures utilizing diverse neuroimaging techniques. Moreover, especially relevant in the clinical environment is finding technical approaches that could be applied to individual participants and not only for group analysis. A 64-channel electroencephalographic recording (EEG) was made with thirty participants divided into three groups of equivalent size (N = 10) (healthy control, low-EDSS (1–2.5) and moderate-EDSS (4–6)). Correlation analysis was applied to multiple measures: behavior, neuropsychological tests (Paced Auditory Serial Addition Test, 3 seconds (PASAT-3s) and the Symbol Digit Modality Test (SDMT)), Expanded Disability Status Scale (EDSS), even-related potential (P3) and event-related desynchronization (ERD) parameters and the correlation scores between individual participant’s P3/ERD maps and the healthy grand average P3/ERDmaps. Statistical analysis showed that diverse parameters exhibited significant correlations. A remarkable correlation was the moderate score found between SDMT and EDSS (r = −0.679, p = 0.0009). However, the strongest correlation was between the value of integrated measures (reaction time, P3 and ERD latency) and EDSS (r = 0.699, p = 0.0006). In regard to correlations for grand average maps between groups, the P3 component exhibited a lower score according to a more deteriorated condition (higher EDSS). In contrast, ERD maps remained stable with an increase of EDSS. Lastly, a Z-transformation of individual values of all variables included in the study exhibited heterogeneity in cognitive alterations in the multiple sclerosis participants.

## Introduction

Understanding the functional and anatomical changes related to cognitive impairment in multiple sclerosis is currently still a challenge. The relatively stochastic localization of lesions in multiple sclerosis (MS) causes complex patterns of cognitive impairment mainly related to slowed cognitive processing speed and episodic memory decline as well as to executive function, verbal fluency and visuospatial analysis [1]. Considering this heterogeneity in the course of cognitive disability, one primary aim is to define protocols and techniques that would allow reliable evaluation of altered brain activity in individual patients.

One possibility for studying the functional alterations of brain activity associated with cognitive impairment is through cognitive tasks (i.e., visual oddball). As a result of this application, diverse behavior variables (reaction time and accuracy) and physiological activity (electroencephalography (EEG)) can be measured. Diverse approaches can be used in the analysis of the EEG signal: time domain (i.e., event-related potentials (ERPs)) or frequency domain (i.e., event-related desynchronization (ERD)). In the particular case of multiple sclerosis, both approaches have evidenced alterations related to cognitive impairment. For instance, the P3 component (as a typical ERP measure) has shown that amplitude could be decreased in MS patients [2, 3, 4, 5, 6, 7], increased in its latency [8, 9, 10, 11, 12], or both simultaneously [13, 14, 15]. All of these modulations were obtained with diverse cognitive paradigms that suggest a non-sensory modality-dependent process representing a more central cognitive function.

With regard to the frequency domain, one possible technique is the ERD (event-related desynchronization) that was described by Pfurtscheller and Aranibar [16] and allows for the analysis of spectral modulations in the millisecond range for the EEG signal. This time-frequency technique allows for observing modulations in brain activity that are not visible in the time domain of ERPs. In the particular case of MS, Leocani et al.[17] looked for changes in ERD related to fatigue and motor dysfunction in MS patients. The results showed that the onset latency of the ERD was not different between experimental groups (fatigued and non-fatigued patients);however, a more widespread anterior topography was found in fatigued patients. Later, Leocani et al. [18] demonstrated a delay in the ERD onset latency that was related to the lesion load presented in MS patients but without correlation to clinical disability.

On the other hand, disability in MS is usually assessed by EDSS (Expanded Disability Status Scale) [19]. The EDSS comprises the evaluation of 8 functional systems (Pyramidal, Cerebellar, Brain Stem, Sensory, Bladder-Bowel, Visual, Cerebral or Mental and Other) in the patient and can assess the progression of the disease. A long time ago, diverse studies have tried to relate EDSS to cognitive measures, in some cases with promising results [9, 13] and in others failing to confirm that link [6, 20, 21]. The progression of disability in MS defined by the EDSS score could serve as a reference for the course of cognitive alterations in patients. An interesting aim would be to define how psychophysiological variables (such as P3 and ERD) evolve through different phases and are related to the EDSS score. In particular, it would be interesting to describe a more general path for cognitive deterioration in MS and its subtypes but at the same time be able to study individual courses of impairment in MS.

For this to be done, not only behavioral parameters (i.e., reaction time in cognitive tasks or neuropsychological scores) but also neuroimaging techniques will be necessary to understand the functional neuroanatomy of cognitive impairment in MS. Indeed, some recent studies have evinced that some EEG parameters (topographical microstates) could be highly correlated to the EDSS score [22] and with cognitive processes.

One of the main goals of the present study is to measure alterations in diverse behavioral and EEG parameters (reaction time, P3 component and alpha-ERD) in two different samples of patients (low and moderate EDSS scores) compared to a healthy sample. A cognitive task (visual oddball) will be used because of its reliability in all of these measures as has been previously tested [23, 24]. It is worth noting that not only a group analysis will be performed for all of these parameters but also individual participant analysis will be performed to contrast the general pattern (group analysis) with individual profiles assessed by individual z-scores. A special consideration will be focusing on checking for individual and group topography alterations related to a higher disability status in these patients as one of the relevant parameters of P3 and ERD measures.

A second main goal is to look for statistically significant correlations between different types of variables (behavior, neuropsychological and neurophysiological) and disability of the MS patient assessed by EDSS. With this aim, we tried to identify neurophysiological parameters that are part of the mechanisms related to the cognitive impairment manifested by MS patients.

We hypothesized that a more severely disabled condition in MS patients will show diverse cognitive alterations such as delays in reaction times, or changes in latency, amplitude and topography of P3 and ERD between healthy and patient groups. Another prediction is that correlation analyses will show a moderate correlation between EDSS and SDMT or the PASAT test as has been described in previous studies. However, we expect that a combination of diverse behavioral and neurophysiological parameters could correlate even better with EDSS score.

Lastly, and as a more general hypothesis, we expect that the moderate-EDSS group will show a larger number of alterations in diverse parameters (Z-scores >1) than that of the low-EDSS group, indicating a more severe cognitive impairment. At the same time, the full set of individual patients will provide evidence of a complex landscape of alterations in all experimental parameters, indicating diverse cognitive status.

## Method

### Participants

Thirty participants were divided evenly into three groups. Two pathological groups were defined by the disability of patients measured through EDSS [18] (low-EDSS (1–2.5) and moderate-EDSS (4–6)). A healthy group with ten participants was also recruited with sociodemographic variables matched between the groups. The participants’ sociodemographic data can be seen in Table 1. All participants were recruited in an MS Unit of the Hospital Universitario Virgen Macarena and exhibited a definite diagnosis of relapsing-remitting multiple sclerosis (RRMS) according to the Poser criteria [25]. Exclusion criteria included the following: forms of MS other than RRMS, suffering a clinical relapse 30 days before participating in the study, EDSS score over 6, presence of comorbid neurodegenerative or psychiatric disorders, history of drug abuse, neurological conditions as head trauma, vascular diseases and seizures, severe depression, significant upper limb impairment, and visual acuity or field deficits. Healthy controls were students and professionals from the Psychology faculty. Exclusion criteria for healthy controls were the same as used for patients in regards to a clean neurological report and checks for visual acuity or field deficits. Beck Depression Inventory (BDI-II) [26, 27] was used to assess symptoms of depression and compare low- and moderate-EDSS groups.

**Table 1:** Demographic data of experimental subjects.

	Low EDSS patients (n = 10)	Mod EDSS patients (n = 10)	Healthy controls (n = 10)	Significance
**Sex (m/f)**	8/2	5/5	6/4	p = 0.360
**Age (years, mean ± SD)**	41 (9.91)	41.9 (10.32)	41.6 (10.12)	p = 0.990
**Handedness (left/right-handed)**	1/9	1/9	1/9	p = 1.00
**Secondary education (yes/no)**	8/2	9/1	10/0	p = 0.874

Key: m: male; f: female; EDSS: Expanded Disability Status Scale; SD: standard deviation; NS: Non-significant difference (p>0.05).

The current study was carried out in compliance with the Helsinki Declaration. All participants were informed about all relevant information in their participation of the study and have at least seven days to decide if they want to be participants and signed informed consent. The experimental protocol and informed consent procedure was reviewed and approved by the ethics committee of the University of Seville (project code: PSI2016-78133-P).

### Neuropsychological assessment

Previous to the ERP study, neuropsychological testing of the patients was provided by well-trained neuropsychologists blinded to the study goals. Diverse cognitive processes such as attention, concentration and speed of information processing were measured through the Paced Auditory Serial Addition Test, 3 seconds (PASAT-3s) [28, 29], and the Symbol Digit Modality Test (SDMT) [30, 31], and were compared to the normative scores described by Sepulcre et al. [32]. Patients were cognitive preserved (some participants of low EDSS group) or mildly impaired (low and moderate EDSS group). In any case, all participants were provided with details about the safety of the experiment and the right to voluntarily withdraw from participation in the presence of a neurologist who confirmed that all participants were able to understand the details about the study and any risk implied.

### Cognitive neurophysiology task

EEG parameters were recorded using a visual oddball paradigm that consisted of the discrimination of uncommon visual stimuli in a sequence of frequent stimuli. A total of 200 trials were used: 50 were target and 150 standard stimuli displayed in a pseudorandom presentation. The target stimulus was a rectangle with a checkerboard pattern comprising red and white squares. The standard (frequent) stimulus was equivalent in size with the same pattern but with black and white squares. Both stimuli were shown in the same position in the center of the screen. A fixation point was present when no stimuli were displayed to prevent changes in eye position during the experiment. The screen was located 70 centimeters from the participant's eyes, and the size of both stimuli was 7.98 of visual angle on the X axis and 9.42 on the Y axis. Both stimuli were presented for 500 milliseconds (ms), and the stimulus onset asynchrony (SOA) was 1.5 seconds during which the participant could respond. The participants were required to press the mouse button with the right index finger when a target stimulus appeared but to ignore the standard stimulus. At the end of the experimental session, reaction time and accuracy percentage were calculated.

### EEG data acquisition and processing

**Fig 1:**
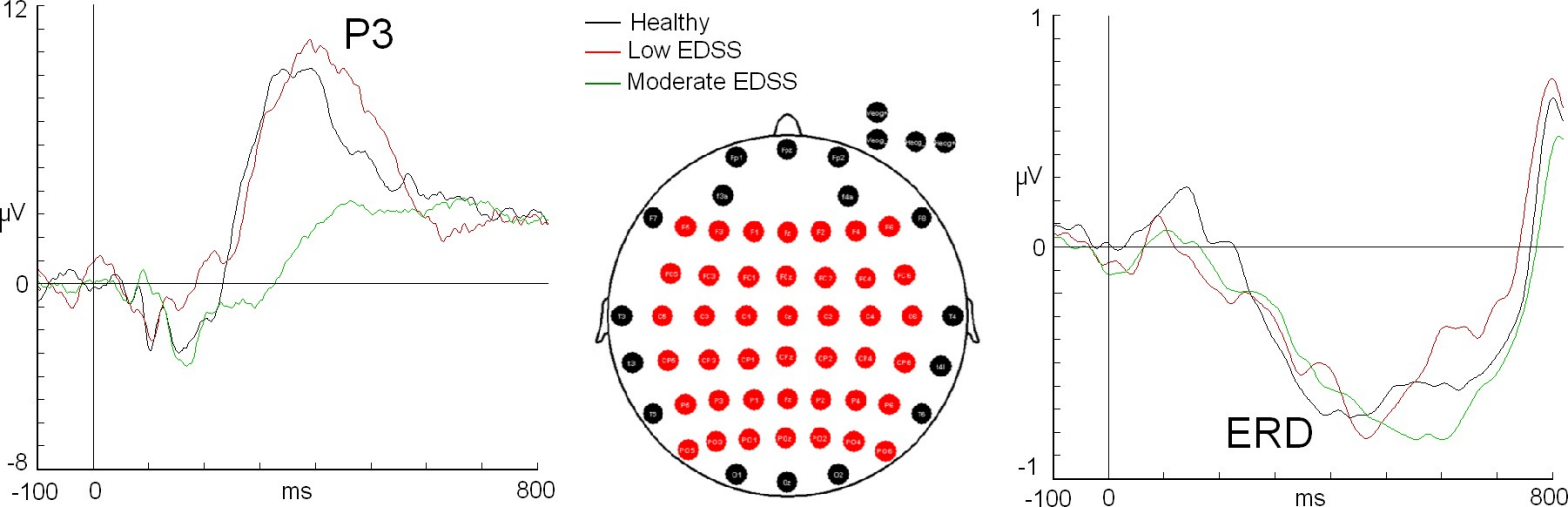
Electrode array and Pzp wave for alpha event-related desynchronization and P3 component. The X axis represents “time” expressed in milliseconds (ms), and the Y axis represents the “amplitude” of the ERP/ERD in microvolts (μV). The vertical dashed line indicates the onset of the stimulus. The black trace corresponds to the healthy group: red corresponds to the low-EDSS group, and green corresponds to the moderate-EDSS group. Note the delayed latency in ERD latency for the moderate-EDSS group compared to healthy control group.

The EEG signal was recorded from 64 electrodes mounted in an elastic cap (Electro-cap) according to the International 10–20 system (Fig 1). All electrodes were referenced during the recording to the linked earlobe channel and offline re-referenced to an averaged reference. Vertical and horizontal electrooculograms (VEOG and HEOG) were recorded with bipolar recordings from electrodes situated in the inferior and superior positions of the right orbit and in the external canthi of the ocular orbits, respectively. The EEG was amplified with a BrainAmp 64-channel system (Brain Products GmbH, Germany) with band limits of 0.01–100 Hz, and a sampling rate of 500 Hz was used. The impedance was kept below 5 kOhms in all derivations used. Previous to the calculation of P3 and ERD parameters, a protocol was applied to the EEG data: ocular correction of the blinking artifact in the scalp electrodes using the algorithm developed by Gratton et al. [33], segmentation of the continuous EEG recording (-100 to 800 ms, zero being the onset of the target stimulus), baseline correction based on the previous interval to the stimulus (-100 to 0 ms), and visual review of EEG epochs and rejection of artifacts.

An automatic rejection protocol was applied, and limits to exclude trials were set as ±75 μV that reject ocular movements performed during the experiment, as well as large muscle activity (i.e., from neck, face, back or jaw muscles). Lastly, averages (with an equivalent number of trials for both P3 and ERD) were calculated for the target stimulus and for each participant. As recommended by Polich [34], all of the individual averages comprised at least 15 artifact-free trials with correct behavior (mean number of trials per group: 30.7 for healthy, 30.4 for low-EDSS and 30.6 for moderate-EDSS). No significant differences were found for the number of trials accepted for averaging among groups.

BrainVision Analyzer Software was used to calculate amplitude, latency and topographical maps for both P3 and ERD modulations. The latency and amplitude values of the P3 component were calculated in the electrode that showed the maximum amplitude for each participant. The criterion to identify the P3 component was to select the maximum positivity from electrodes of a matrix of 6 x 7 (Fig 1) (in this case, the Pzp derivation) in the interval between 300 and 450 milliseconds. After the latency was determined by the maximum amplitude, amplitude values for the rest of the electrodes (matrix 6x7, see Fig 1) were exported in the same latency for topographical study, as some authors suggest [35].

Alpha event-related desynchronization (ERD) was obtained from the EEG signal applying the following protocol: 1) filtering in the 8–13 Hz band, 2) rectifying (which turns negative voltage values into positive values) and 3) averaging [16, 36]. Latency and amplitude values of the alpha ERD were calculated in the electrode that showed the maximum amplitude (as with P3, the Pzp derivation). The alpha “valley” was identified as the maximum negativity in the interval between 250 and 700 milliseconds. Calculation of voltage values were performed in regards to the baseline previous to the onset of the stimuli (−100 to 0 ms) for both P3 and ERD.

### Statistical analyse

Statistical analyses were performed with SPSS v20 software. Considering the limited number of cases for each group (N = 10), a nonparametric test (Kruskal-Wallis) was conducted to determine potential differences in the behavioral and neurophysiological variables (reaction time, accuracy and ERD and P3 latencies). In all cases, pairwise comparisons were performed with a Bonferroni correction for multiple comparisons. Adjusted p-values are presented in the results section. The transformation of all parameters into a z-score was calculated using the following formula: Z = (value-mean)/standard deviation. Mean and standard calculations were obtained from the healthy group.

To analyze the correlations between EDSS and all behavioral and EEG parameters (ERD and P3 latencies and map scores), Pearson’s product-moment r was employed. Neuropsychological data were only correlated with other measures for patient groups. Data from both patient groups were combined to perform the correlation analyses.

In addition, we defined two variables termed “map scores” (for P3 and ERD) that were calculated as the correlation score between the voltage from the scalp electrodes (matrix 6x7 referred to above) for each patient and the corresponding voltage values of the grand average P3/ERD maps from the ten healthy controls. These two new variables were also included in the variables that were correlated to the EDSS score. Finally, we calculated the mean of a combined measure of three z-scores from three variables (reaction time, P3 latency and ERD latency) and termed it as “Z3”.

As a general consensus, p<0.05 was considered statistically significant. However, as recommended by Kileny & Kripal [37], a new level of significance was determined by the classic value (0.05) divided by the number of correlations performed (25) (the new p level was set to 0.002).

## Results

### Behavior

A Kruskal-Wallis test was conducted to determine whether there were differences in the reaction time between the experimental groups: "healthy control" (n = 10), "low-EDSS" (n = 10), and "moderate-EDSS" (n = 10). The median (Mdn) RT values were significantly different between the different levels of experimental groups, χ2(2) = 6,265, p = .043. The post hoc analysis indicated statistically significant differences in the RT values between the healthy control group (Mdn = 10,3) and the moderate-EDSS group (Mdn = 20,1) (p = .038). There were no significant differences between the other comparisons. The faster value corresponded to the healthy control group (418 ± 72.32 milliseconds (ms)), followed by the low-EDSS group (457 ± 69.19 ms) and finally the longer value belonged to the moderate-EDSS group (494 ± 66.40 ms) (Table 2). Accuracy variables assessed by Kruskal-Wallis test showed no statistical significance between groups (p = 0.984) (healthy: 99.3 ± 0.97, low-EDSS: 99.3 ± 0.71 and moderate-EDSS: 99.2 ± 1.34). In the case of transformation of individual reaction time values in z-scores, one participant from the healthy group, three for low-EDSS, and five from moderate-EDSS exhibited values higher than 1.

**Table 2:** Individual behavioral, neuropsychological and EEG parameters in three experimental groups.

*Group and subjects*	*RT*	*RT Z*	*P3 Lat*	*P3 Lat Z*	*ERD Lat*	*ERD Lat Z*	*Map P3*	*Map ERD*	*EDSS*	*SDMT*	*PASAT*	*BECK*
***HC1***	**408**	**-0,13**	**316**	**-0,93**	**460**	**0,18**	**0,76**	**0,962**				
***HC2***	**400**	**-0,25**	**322**	**-0,83**	**384**	**-0,67**	**0,967**	**0,945**				
***HC 3***	**594**	2,45(*)	**406**	**0,59**	**486**	**0,47**	**0,745**	**0,847**				
***HC 4***	**356**	**-0,86**	**396**	**0,42**	**634**	2,13(*)	**0,762**	**0,834**				
***HC 5***	**377**	**-0,56**	**504**	2,25 (*)	**432**	**-0,13**	**0,694**	**0,759**				
***HC 6***	**350**	**-0,95**	**366**	**-0,08**	**488**	**0,49**	**0,835**	**0,925**				
***HC 7***	**396**	**-0,30**	**350**	**-0,36**	**436**	**-0,09**	**0,889**	-0,048(*)				
***HC 8***	**400**	**-0,25**	**404**	**0,56**	**464**	**0,22**	**0,616**	-0,386(*)				
***HC 9***	**485**	**0,93**	**318**	**-0,90**	**306**	**-1,55**	**0,684**	**0,812**				
***HC 10***	**410**	**-0,11**	**330**	**-0,69**	**350**	**-1,06**	**0,682**	**0,925**				
***HC GLOBAL***	**418**		**371**		**444**		**0,763**	**0,658**				
***HC STD***	**72,32**		**58,70**		**89,53**		**0,11**	**0,47**				
	**RT**	**RT Z**	**P3 Lat**	**P3 Lat Z**	**ERD Lat**	**ERD Lat Z**	**Map P3**	**Map ERD**	**EDSS**	**SDMT**	**PASAT**	**BECK**
***LOW1***	**501**	1,15(*)	**378**	**0,12**	**676**	2,61(*)	0,461(*)	0,409(*)	**2**	**36**	**52**	**15**
***LOW2***	**439**	**0,28**	**410**	**0,66**	**458**	**0,16**	**0,876**	0,077(*)	**2**	**76**	**54**	**6**
***LOW3***	**414**	**-0,06**	**272**	**-1,68**	**406**	**-0,43**	**0,85**	**0,883**	**2**	**42**	**44**	**20**
***LOW4***	**456**	**0,52**	**374**	**0,05**	**474**	**0,34**	0,438(*)	-0,639(*)	**2**	**48**	**56**	**9**
***LOW5***	**334**	**-1,17**	**348**	**-0,39**	**424**	**-0,22**	**0,865**	**0,871**	**1,5**	**45**	**43**	**8**
***LOW6***	**463**	**0,62**	**390**	**0,32**	**508**	**0,72**	**0,791**	**0,872**	**2**	28(*)	17(*)	**13**
***LOW7***	**553**	1,88(*)	**334**	**-0,63**	**460**	**0,18**	**0,755**	0,398(*)	**2,5**	**39**	**47**	**20**
***LOW8***	**553**	1,87(*)	**300**	**-1,20**	**446**	**0,02**	**0,77**	**0,921**	**1**	**50**	**42**	**15**
***LOW9***	**471**	**0,73**	**422**	**0,86**	**564**	1,35(*)	0,413(*)	0,235(*)	**2**	**53**	**51**	**14**
***LOW10***	**386**	**-0,45**	**402**	**0,53**	**572**	1,44(*)	0,598(*)	**0,749**	**2**	**49**	**43**	**7**
***LOW GLOBAL***	**457**		**363**		**499**		**0,682**	**0,478**	**1,9**	**46,6**	**44,9**	**12,7**
***LOW STD***	**69,19**		**48,99**		**82,91**		**0,19**	**0,50**	**0,39**	**12,76**	**11,02**	**5,08**
	**RT**	**RT Z**	**P3 Lat**	**P3 Lat Z**	**ERD Lat**	**ERD Lat Z**	**Map P3**	**Map ERD**	**EDSS**	**SDMT**	**PASAT**	**BECK**
***MOD1***	**453**	**0,49**	**316**	**-0,93**	**484**	**0,45**	0,469(*)	0,437(*)	**4**	**50**	**45**	**10**
***MOD2***	**560**	1,97(*)	**492**	2,05(*)	**570**	1,42(*)	**0,62**	0,624(*)	**6**	13(*)	**56**	**18**
***MOD3***	**527**	1,51(*)	**596**	3,81(*)	**594**	1,69(*)	0,299(*)	**0,821**	**6**	20(*)	**52**	**3**
***MOD4***	**467**	**0,68**	**298**	**-1,24**	**534**	1,01(*)	**0,782**	**0,948**	**4**	**50**	**46**	**21**
***MOD5***	**475**	**0,79**	**590**	3,71(*)	**516**	**0,81**	**0,867**	**0,727**	**5**	**32**	22(*)	**16**
***MOD6***	**631**	2,96(*)	**532**	2,73(*)	**540**	1,08(*)	0,542(*)	**0,789**	**5,5**	29(*)	22(*)	**16**
***MOD7***	**436**	**0,25**	**360**	**-0,19**	**610**	1,87(*)	0,56(*)	**0,872**	**5,5**	**32**	**41**	**30**
***MOD8***	**398**	**-0,28**	**406**	**0,59**	**634**	2,13(*)	0,543(*)	**0,903**	**4**	**42**	**51**	**20**
***MOD9***	**494**	1,06(*)	**558**	3,17(*)	**610**	1,87(*)	0,243(*)	**0,94**	**6**	20(*)	**42**	**20**
***MOD10***	**498**	1,11(*)	**628**	4,36(*)	**584**	1,57(*)	0,521(*)	**0,919**	**4**	26(*)	18(*)	**11**
***MOD*** ***GLOBAL***	**494**		**478**		**568**		**0,545**	**0,798**	**5**	**38,15**	**40,91**	**16,5**
***MOD*** ***STD***	**66,40**		**123,03**		**47,75**		**0,19**	**0,16**	**0,91**	**12,64**	**13,81**	**7,34**

Abbreviations. RT: Reaction time (in milliseconds).Lat: Latency (in milliseconds, ms). ERD: Event-related desynchronization. Z: Z-transformation for the variable.Mod-EDSS: Moderate-EDSS. Asterisks (*) indicate in Z-columns (RT Z, P3 Lat Z and ERD Lat Z) values higher than 1 for z-score. Asterisks in Map columns specify correlation scores lower than 0.6 for P3 and 0.7 for ERD.

### Neuropsychological data

Table 2 presents the SDMT and PASAT scores for individual patients. Using the normative values from Sepulcre et al. [29], it is possible to observe that low-EDSS patients were cognitively unimpaired (except in one case). In contrast, five of the moderate-EDSS group showed lower hits for one or both tests that were under the cut-off value for their age and education level.

### Alpha event-related desynchronization (ERD)

**Fig 2:**
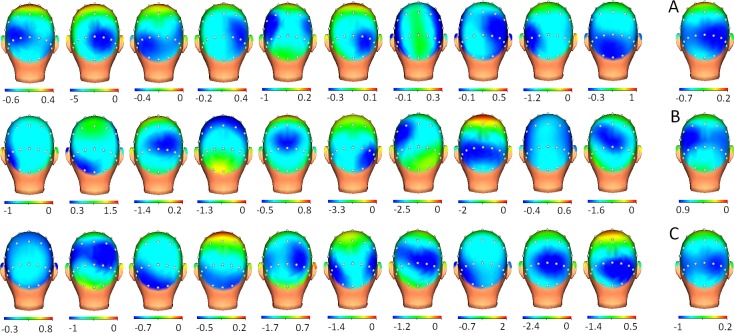
Grand average and individual 3D head maps of ERD (event-related desynchronization) for healthy, low- and moderate-EDSS groups. 3D head maps are displayed for each of the 10 participants of each group (upper line: healthy group; midline: Low EDSS group; bottom line: Moderate EDSS group) and the grand average for each group (A): healthy; (B): Low EDSS and (C) Moderate. Note that GA topographies were similar between groups.

Potential differences among the three groups ("healthy control", "low-EDSS" and "moderate-EDSS") in the ERD latency were examined using a Kruskal-Wallis test. The median ERD latency values were significantly different between the different levels of experimental groups, χ2(2) = 10,095, p = .006. The post hoc analysis indicated statistically significant differences in the ERD values between the healthy control group (Mdn = 10,1) and the moderate-EDSS group (Mdn = 22,3) (p = .005) (Fig 1 and Table 2). There were no significant differences between the other comparisons. In regards to the amplitude, no differences were exhibited by Kruskal-Wallis test (p = 0.968) (healthy: −0.864 ± 1.78 μV, low-EDSS: −1.010 ± 0.97μV and moderate-EDSS: −0.885 ± 0.62 μV) (see Fig 2). In the individual analysis for latency parameter, one participant from the healthy, three from low-EDSS and eight in the moderate-EDSS groups exhibited a z-score higher than 1 (Table 2).

### Event-related potentials (P3)

**Fig 3:**
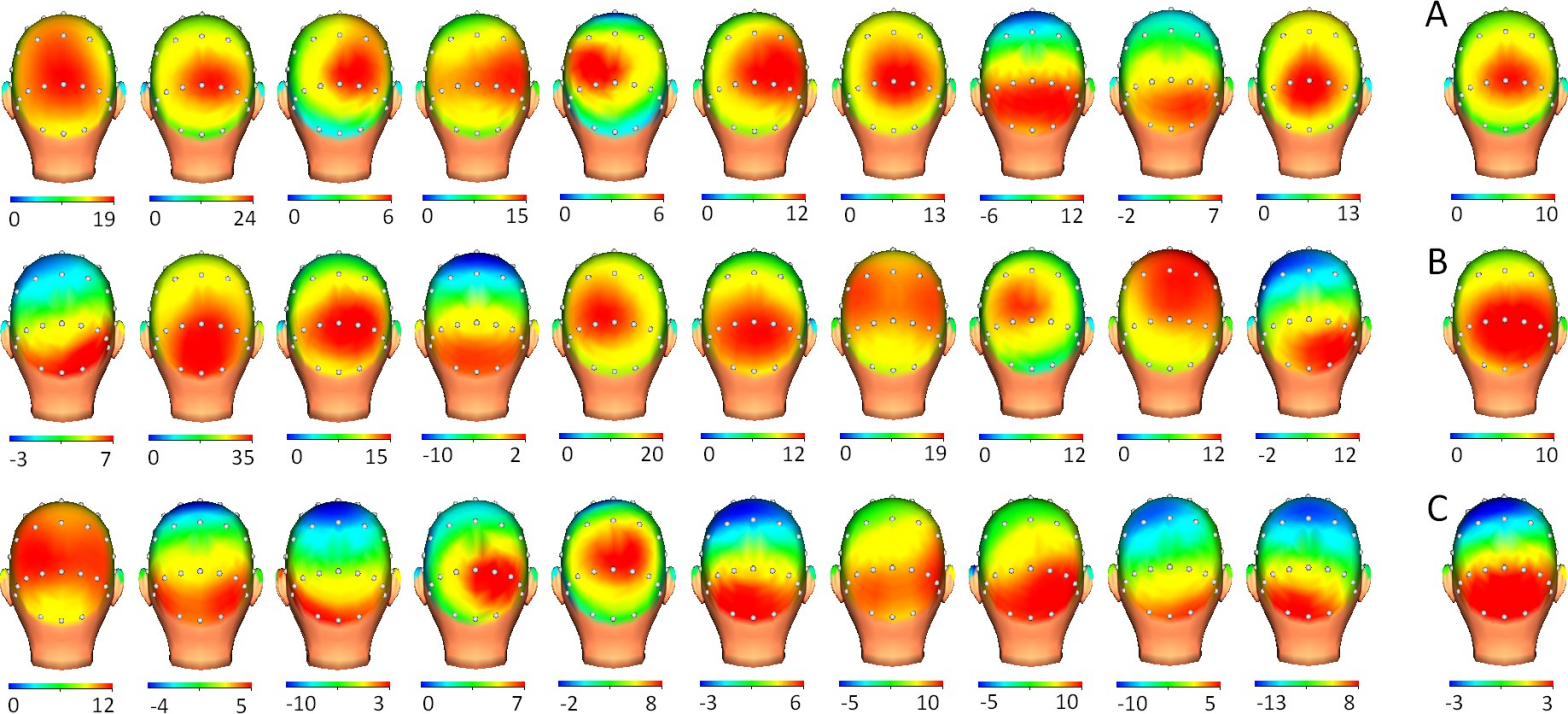
Grand average and individual 3D head maps of P3 for healthy, low- and moderate-EDSS groups. 3D head maps are displayed for each of the 10 participants of each group (upper line: healthy group; midline: Low EDSS group; bottom line: Moderate EDSS group) and the grand average for each group (A): healthy; (B): Low EDSS and (C) Moderate. Note that the topography for GA is moved to more posterior areas as disability (EDSS) increases.

The Kruskal-Wallis test did not show significant differences among the groups for the P3 latency. Despite the lack of significant differences, healthy (371 ± 58.70 ms) and low-EDSS (363 ± 48.99 ms) groups were faster in their P3 latency compared to the moderate-EDSS group (478 ± 123.03 ms) (Fig 1). In the case of the amplitude, no statistically significant differences were found between groups for this variable (p = 0.105) (healthy (11.22 ± 5.39 μV), low-EDSS (12.74 ± 9.09 μV) and moderate-EDSS (6.78 ± 2.33 μV)) (Fig 3). In the individual analysis of P3 latencies, there was not a single participant from the low-EDSS group that exhibited a z-score higher than 1 in contrast to six patients in the moderate-EDSS group (Table 2).

### Correlation analyses

**Fig 4:**
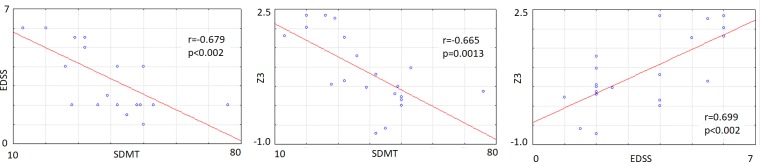
Scattergrams for the significant correlations between EDSS, SDMT and Z3 variables. Abbreviations:Z3: Z-score of combined variables (reaction time, P3 latency and ERD latency), SDMT:Symbol Digit Modality Test and EDSS: Expanded Disability Status Scale.

After the analyses, only three correlations showed a statistically significant value (p<0.002): SDMT vs EDSS (r = −0.679, p = 0.0009); SDMT vs Z3, (r = −0.665, p = 0.0013) and EDSS vs Z3 (r = 0.699, p = 0.0006) (see Fig 4). A statistical trend was observed for P3 latency and the EDSS variable, r = 0.638, p = 0.0024).

In the correlation analyses for P3 maps between groups, the healthy and low-moderate group exhibited a good score (r = 0.873, p<0.002), but a lower score was found when the comparison was made between healthy and moderate-EDSS groups (r = 0.524, p<0.002). In the case of ERD maps, correlation scores between healthy and patient groups were in both cases at an excellent level (healthy vs low-EDSS, r = 0.956, p<0.002; healthy and moderate-EDSS, r = 0.976, p<0.002). Amplitudes for P3 and ERD were not included in these analyses due to the absence of significant differences across the three groups.

## Discussion

The reaction time showed a significant difference between the healthy control and moderate-EDSS groups. This difference was based on the slower responses for the moderate-EDSS group. This result suggests progressive deterioration in cognitive processing indexed by a behavioral variable in the moderate-EDSS group and an intermediate status for the low-EDSS group. It is necessary to note that the differences in reaction time were not a product of compensation for lower accuracy (speed-accuracy tradeoff) because there were no significant differences for the accuracy variable between groups.

Finally, transformation of the reaction time variable in z-scores for each participant revealed that the moderate-EDSS group has a higher number of individuals with more than 1 (5) and three cases for low-EDSS. This result suggests that the reaction time in an oddball visual task can provide relative evidence of progressive cognitive deterioration in RRMS patients.

The Pearson’s product-moment r between EDSS and reaction time exhibited a poor correlation score for these variables (r = 0.393, p>0.05), indicating that behavior by itself is not very representative of disability progression. Moreover, it is necessary to mention that the healthy group also showed one individual with a z-score higher than one, indicating the possibility of false positives with this variable. In any case, it is necessary to remark that changes in the reaction time for MS groups could also be due to motor impairment and not only sensory and central cognitive impairments. Moreover, a global EDSS score could hide a potential motor impairment in an MS sample. Future studies considering subscores and peripheral and central motor evaluations would be required to confirm this possibility.

The main result for neuropsychological variables is that the low-EDSS group was almost totally unimpaired as assessed by the SDMT and PASAT tests and considering normative scores by Sepulcre et al. [32]. Five of the moderate-EDSS patients exhibited SDMT/PASAT scores under the cut-off value defined by Sepulcre et al.[32]. It seems reasonable to suggest that the progression in the disability of patients is related to cognitive impairment as has also been confirmed by the significant correlation found between SDMT and EDSS (r = -0.679, p = 0.0009).

Statistical analysis of alpha ERD latency evinced that the moderate-EDSS group showed a significant delay compared to the healthy and low-EDSS groups. This result is in line with other described spectral alterations in MS patients. Leocani et al.[18] showed delays in the mu-ERD onset latency suggesting a disruption in the functional cortico-cortical and cortico-subcortical connections in MS patients during programming voluntary movement. In line with Leocani et al. [18], a mildly impaired ERD latency in the moderate-EDSS group from our study could be a consequence of partial disconnection (demyelinization) between neural elements responsible for alpha ERD modulation. The lack of amplitude or topography changes suggests no neuronal loss or atrophy associated with the delay of the ERD latency. It is noteworthy that the alteration of the alpha ERD latency is present in 8 out of 10 patients from the moderate-EDSS group and in three from the low-EDSS group. It seems reasonable to note that this parameter is related to the disability score of the MS patient.

In the individual alpha ERD maps,diverse results were remarkable for this parameter. First, some patients exhibited low correlation scores for their individual maps compared to the grand average map of the healthy group. Considering that the grand average maps for ERD between groups reached excellent scores (r>0.9), low correlation scores support previous studies in which it was noted that the grand averaging can hide individual particularities [23, 24]. Secondly, the number of low score maps in the low-EDSS group is higher compared to that of the moderate-EDSS group (indeed, healthy and moderate-EDSS groups have the same ratio for low scores maps: 2/10).Third, there is not a perfect match between the alterations seen in topography compared to the changes found in the ERD latency, demonstrating that both variables are not necessarily linked. A possible interpretation of these results could be that the alteration of the ERD topography represents a possible compensatory mechanism active in the low-EDSS group. A study from Kiiski et al.[38], has suggested that the increase of the decrement for ERD could represent a compensatory mechanism. In this case, we suggest that changes in the topography, without changes in latency or amplitude, can index compensatory mechanisms in early EDSS phases of MS patients.

A remarkable result regarding the topography of alpha ERD is that, despite the reliable distribution in grand average maps between groups, individual analyses revealed that a more frontal and sometimes lateralized distribution of ERD modulation is present in the low-EDSS patients compared to that of the healthy grand average. This result is in line with previous studies where a more widespread anterior topography was found in fatigued patients [18]. However, a surprising result is that altered topography in low-EDSS looks like a transitional modulation because only two patients from the moderate-EDSS group showed low correlation scores. In any case, a follow-up study for the low-EDSS group will be necessary to confirm this hypothesis.

In the case of the P3 component, no significant differences were identified for the latency parameter. A correlation between EDSS and P3 latency has been observed in previous studies [9]. However, in previous studies from our group, no relation was found between these variables [6, 11]. Despite the lack of a significant difference in the P3 latency, the progressive increase in the mean values from the healthy controls to the moderate-EDSS supports the hypothesis that the demyelinization process causes a progressive slowing of information processing that is represented by a delayed P3 peak.

The amplitude variable also exhibited a decrease (not statistically significant) in the moderate-EDSS group compared to that of the healthy group and low-EDSS groups. Some studies have described similar decrement in P3 amplitude and have suggested that lack of attentional resources could be behind this modulation [5, 6]. Another possibility suggested by our group is that the reduction of the amplitude is based on the difficulty of synchronizing all of the neural mechanisms involved in the building of P3 and that therefore different time intervals for them caused a flattening of the voltage curve for P3 [24]. However, more studies in the future could confirm if all of these hypotheses are verified.

The third parameter for the P3 component (topography) showed in the grand average maps that the correlation score was diminishing in line with the progression of disease status. A lower P3 map score suggests higher disability. This result supports previous studies in which spontaneous fluctuation topography was related with EDSS [22] or in a high-density EEG detecting topographical differences between MS patients and controls for both early and late components for the visual modality [2]. All of this evidence supports previous studies in which topography is considered a highly reliable parameter for ERP components that allow for an individual analysis of cognitive processing [24, 39, 40].

Although no individual P3 latency was higher than z>1 for low-EDSS patients, some topography maps in this group exhibited a low P3 map score (4 out 10). This result suggests that P3 latency is probably a highly specific parameter for assessing the cognitive course in MS patients; however, changes in the topographical distribution could suggest that neural generators are affected in their orientation (i.e., caused by atrophy) even before cognitive impairment is visible by other P3 parameters (latency and amplitude).

In other correlation analyses, P3 latency exhibited a moderate score with EDSS (r = 0.638, p = 0.002). However, the higher score was found for Z3 (combination of z-scores of reaction time, P3 latency and ERD latency) (r = 0.699, p = 0.0006), suggesting that the combination of all of these parameters related with cognitive processing correlate at an acceptable level with the disability status (EDSS) in these patients.

The general pattern that is represented in the current data is that several parameters related with cognitive processing (behavioral and EEG) are altered at a more advanced phase of disability of MS patients (moderate-EDSS group). However, individual analyses reflect that, in the low-EDSS phase of the disease, diverse cognitive measures could be altered and provide,with just a few cognitive variables, a complex picture of possible compensatory mechanisms or cognitive processes that are not yet altered but will be once reaching moderate-EDSS status. Indeed, it is possible to find dissociation between behavioral parameters (reaction time) and EEG parameters (P3 and ERD latency) in individual patients who support previous studies in healthy participants [41].

It is necessary to note that the healthy grand average map is valid for a specific cognitive task [42] and that if there is a purpose for studying cognitive alterations in patients, calculating the healthy grand average map would be required for every task implemented [23, 24]. A limitation that has to be considered is that the sample size in the present study is limited (n = 10 for all groups) and that a larger sample size would be recommended for more accurate detection of cognitive alterations in MS patients. Nevertheless, it is also noteworthy that such a small sample size was able to determine high correlation scores in topographical maps and highly statistically significant differences for P3/ERD latency in the moderate-EDSS group. Moreover, individual z-score analyses have shown a reasonable ratio of false positives considering critical variables including reaction time or P3 and ERD latencies.

Future research using different approaches to group vs individual analyses, and other research that uses machine learning to find hidden patterns in the cognitive status and evolution [43, 44] are required for a better understanding of cognitive alterations suffered by neurological patients and the possibility of defining therapeutic targets for treatment tailored to every individual progression of the disease.

In conclusion, the present study supports the idea of a moderate correlation between disability assessed by EDSS and alterations in diverse parameters related to cognitive processing. Interestingly, a general pattern is observed with no changes in the P3 latency/amplitude in the early phases of the disease but with an altered topography (even more for moderate-EDSS) with regard to the healthy grand average map. Alpha ERD latency is also affected in moderate-EDSS patients but without changes in topography between groups. However, based on the individual analyses, a complex landscape of diverse alterations in cognitive elements and their chronology may be present in the progression of MS. Further research with a higher number of samples and diverse forms of MS are necessary to enhance our knowledge regarding the functional neuroanatomy basis of cognitive impairment in MS.
